# Myoclonic epilepsy with ragged-red fibers: A case report

**DOI:** 10.3892/etm.2014.2140

**Published:** 2014-12-16

**Authors:** XUE-FAN YU, JING MIAO, YAN LI, XIN-MEI JIANG, YU-GANG MA, HONG-MEI MENG

**Affiliations:** Department of Neurology, The First Hospital of Jilin University, Changchun, Jilin 130021, P.R. China

**Keywords:** myoclonia, epilepsy, mitochondrial encephalomyopathy

## Abstract

Myoclonic epilepsy with ragged-red fibers is a maternally inherited disease that is characterized by myoclonic epilepsy, cerebellar ataxia and progressive muscular weakness. The present study reports the case of a 25-year-old male who presented with paroxysmal left upper limb tics and weakness for two years. Neurological examination revealed intact cranial nerves, decreased deep tendon reflexes and decreased sensation of touch, pain and vibration. The gait of the patient was broad and he was unable to walk in a straight line. Local cortical atrophy was also observed in the left temporal-occipital cortex on a magnetic resonance imaging scan. The muscle biopsy revealed ragged-red fibers. Therefore, the present study hypothesized that imaging observations and follow-up examinations are important in patients with myoclonic epilepsy.

## Introduction

Myoclonic epilepsy with ragged-red fibers (MERRF) is a neurological disorder that is characterized by muscle twitches, weakness and progressive stiffness that affects numerous muscles of the body. Patients with MERRF can additionally exhibit recurrent seizures, difficulty coordinating movements, peripheral neuropathy and the slow deterioration of intellectual function (1). MERRF is a maternally inherited mitochondrial encephalomypathy caused by a mtDNA mutation. The most common mutation is the m.8344A>G mutation in the mtDNA gene, MT-TK, which encodes mitochondrial transfer (t)RNA lysine. The mutation causes poor aminoacylation of the mutant tRNA and premature termination of translation at lysine codons (2), which subsequently decreases the activity of respiratory chain complexes I and IV, as well as the respiration rate and the mitochondrial membrane potential (3). Ragged red fibers consist of a large collection of abnormal-appearing mitochondria (4).

To date, the principal treatment for MERRF is symptomatic. However, standard traditional antiepileptic drug therapy appears to be effective, and coenzyme Q10 is often used in an attempt to improve mitochondrial function (5,6). MERRF is a very rare condition in the Chinese population, as is the 8344A>G mutation (3.9% of all pathogenic mtDNA mutations) (7). The present study reports the case of a 25-year-old male with mitochondrial encephalomyopathy, who presented with myoclonic epilepsy.

## Case report

**Figure 1:**
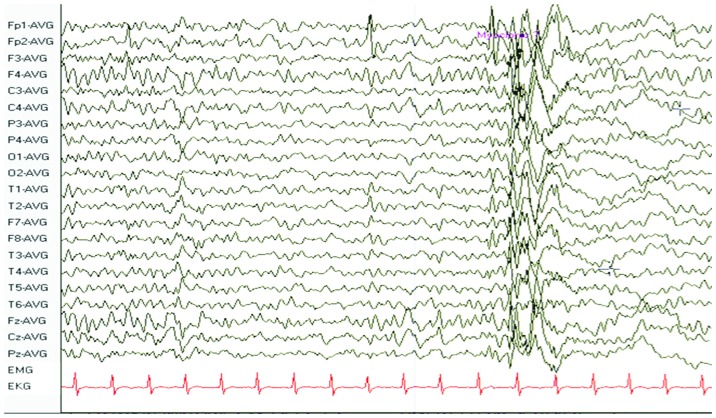
The electroencephalogram showed diffuse spikes and slow waves, predominately in the frontal and temporal lobes.

**Figure 2:**
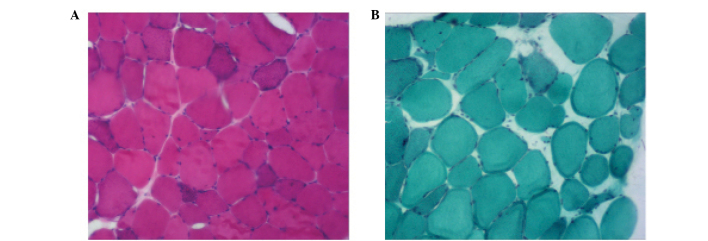
Biopsy of the biceps muscle. (A) Hematoxylin and eosin staining and (B) Gomori’s trichrome staining of the biceps muscle showed variation in fiber size and ragged-red fibers.

A 25-year-old male presented with paroxysmal left upper limb tics and weakness that had been ongoing for two years. The involuntary limb tics exhibited a sudden onset and lasted for seconds, but were not accompanied by consciousness disturbance. The patient had approximately 10 attacks per day, which were accompanied by limb weakness. A magnetic resonance imaging (MRI) scan was performed initially and was found to be normal. The patient had received irregular diazepam administration from the onset of the disease; however, the symptoms became increasingly more serious. The patient was prescribed 600 mg per day valproate sodium on admission to hospital to control the seizures, but experienced one or two attacks per month subsequent to the administration of valproate sodium. The past medical history of the patient was unremarkable. On examination, the patient was alert and his pupils adjusted to light. Neurological examination revealed intact cranial nerves, but decreased deep tendon reflexes and a decreased sensation of touch, pain and vibration. The gait of the patient was broad and he was unable to walk in a straight line. Full strength was observed in all the muscle groups. The results of the Romberg, heel-knee-shin and finger-to-nose tests were normal. An electroencephalogram (EEG) revealed diffuse spikes and slow waves, predominantly in the frontal and temporal lobes (Fig. 1). A further MRI scan was performed and revealed increased signal density on T2-weighted imaging and decreased signal density on T1-weighted imaging in the right temporal occipital cortical lesions. Local cortical atrophy was also observed in the left temporal-occipital cortex. In addition, the lactic acid concentration (5.2 mmol/l) had markedly increased. The results of the carotid ultrasound and electromyography were normal. A biopsy of the biceps muscle demonstrated a variation in fiber size and the presence of ragged-red fibers (Fig. 2). In addition to the prescribed 600 mg per day valproate sodium, the patient was administered 10 mg per day coenzyme Q10 for approximately 2 years. Two years later his symptoms relieved and an EEG showed less spikes and slow waves than it had previously shown.

## Discussion

Mitochondrial encephalomyopathies are a group of disorders characterized by impaired oxidative metabolism, which result in the impairment of skeletal muscles and the central nervous system. The clinical presentation of a mitochondrial encephalomyopathy is highly variable and diagnosis is often difficult, requiring an extensive series of clinical studies and muscle biopsies or DNA testing.

The present case report described a young male with involuntary tics and a diagnosis of MERRF based on EEG findings (8). Notably, the patient also presented with a number of associated symptoms, including weakness and a broad gait while walking. In a previous study (9), abnormal brain MRI observations were reported in patients with mitochondrial encephalomyopathy. The most frequent abnormalities in patients with mitochondrial encephalomyopathy are widespread white matter hyperintensity and supratentorial cortical and cerebellar atrophy. In certain cases (10), brain abnormalities are absent. In the present case, the first MRI scan was normal. The patient subsequently developed right temporal-occipital cortical long T1 and T2 signals in the MRI scans. Local cortical atrophy was also observed in the left temporal-occipital cortex. This observation was consistent with brain MRI abnormalities in patients with mitochondrial encephalomyopathy.

Pathologically, MERRF is characterized by a variation in fiber size and ragged-red fibers. In the present study, the muscle biopsy and pathological findings were consistent with MERRF. Therefore, the present study hypothesizes that imaging observations and follow-up examinations are important for patients with myoclonic epilepsy.
